# Tachinid (Diptera, Tachinidae) parasitoids of *Lobesia botrana* (Denis & Schiffermüller, 1775) (Lepidoptera, Tortricidae) and other moths

**DOI:** 10.3897/zookeys.934.50823

**Published:** 2020-05-19

**Authors:** Pier Luigi Scaramozzino, Filippo Di Giovanni, Augusto Loni, Silvia Gisondi, Andrea Lucchi, Pierfilippo Cerretti

**Affiliations:** 1 Department of Agriculture, Food and Environment, University of Pisa, via del Borghetto 80, 56124, Pisa, Italy University of Pisa Pisa Italy; 2 Department of Biology and Biotechnology “Charles Darwin”, Sapienza University of Rome, Piazzale A. Moro 5, 00185, Rome, Italy Sapienza University of Rome Rome Italy; 3 Natural History Museum of Denmark, Universitetsparken 15, 2100, Copenhagen, Denmark Natural History Museum of Denmark Copenhagen Denmark

**Keywords:** biological control, *Cacoecimorpha pronubana*, *Daphne gnidium*, *Ephestia unicolorella* subsp. *woodiella*, Erebidae, *Euproctis chrysorrhoea*, puparia, *Quercus* spp., *Tortrix viridana*, *Vitis vinifera*

## Abstract

The present paper reports data on the biology of eleven species of tachinid flies collected in Italy and Spain on different host plants and emerged from different host larvae. An annotated list of the eleven species emerged from the collected lepidopterans is provided; information about distribution and biology are given as well as the description of their puparia. Two new parasitoid species of the European Grapevine Moth (EGVM) *Lobesia botrana* were recorded: *Clemelis massilia*, whose host preferences were unknown so far, and *Neoplectops pomonellae*. A list of lepidopteran pest species with their associated plants and tachinid parasitoids is then given in order to highlight the relationships among the three components of the biocenosis (plant, herbivore and parasitoid). Eventually, due to the great economic importance of *L. botrana* in viticulture, a preliminary identification key to the puparia of its tachinid parasitoids is provided.

## Introduction

Every year world agricultural yield is reduced by 10–16% both by pre- and post-harvest pests (Bradshaw et al. 2016). Crop losses caused by arthropods appear to be higher in modern industrial agriculture than in traditional agriculture which employs more environmentally friendly and sustainable practices (Culliney 2014, Lucchi and Benelli 2018). Every year, 35 million hectares of forest, especially in temperate and boreal areas, are damaged by outbreaks of harmful insects (FAO 2010, Kenis et al. 2019). With the increase in the volume and speed of international trade, together with climate change, the incidence of these outbreaks is also increasing (FAO 2010, Kenis et al. 2019). Lepidoptera is one of the main groups of plant feeding insects which can be potentially dangerous to both agriculture and forestry.

Biological control is a sustainable and environment-respectful method used for the containment of harmful insects. Among the biological control agents (BCAs), in most cases parasitoid insects are renowned for their effectiveness and specificity. The main orders of insect parasitoids are Hymenoptera and Diptera. Notwithstanding the great knowledge that has been acquired on this topic, many aspects of the parasitoid behaviour and action towards the host are still awaiting clarification. In this context, taxonomy plays a key role for a better understanding of the species to be used as BCAs, and their relative host range. Sometimes rather large host ranges may be an indication of a poorly investigated parasitoid taxon. In other cases, however, they can be due to inaccurate identifications of both the host or the parasitoid, as often occurs with old reports. Presenting data on host-parasitoid relationships can help verify and possibly confirm old records. Moreover, they also increase the amount of data available for future studies focusing on the host range extension and its possible variation under different regional conditions.

Among Diptera, Tachinidae is a megadiverse family, representing one of the most diverse lineages of parasitoids (Stireman et al. 2019). Despite being a well-investigated family in Europe, some biological aspects, mostly concerning their host-association, are still poorly known (Mückstein et al. 2007). Most of the biological information available so far was recently resumed in the Preliminary Host Catalogue of Palaearctic Tachinidae (Tschorsnig 2017), making comparative studies easier to develop. Among all the tachinid hosts, the European Grapevine Moth (EGVM), *Lobesia botrana* (Denis & Schiffermüller, 1775), represents a key pest in viticulture (Ioriatti et al. 2011); this moth is present in the grape-growing regions of Europe, Near East, southern Russia, northern and western Africa, and it was accidentally introduced in North and South America (Ioriatti et al. 2012, Cooper et al. 2014). The most common wild host plant of EGVM is the spurge flax, *Daphne gnidium* L. (Thymelaeaceae), which possibly represents its native host from which EGVM later expanded towards vineyards (Scaramozzino et al. 2017a). Both grapevine and *D. gnidium* are hosts of other moths, which can be in turn exploited by the same parasitoid community associated with EGVM (Villemant et al. 2012, Scaramozzino et al. 2017b).

In the present paper we report parasitoid-host records for eleven species of tachinids collected in Italy and Spain on EGVM and other lepidopteran hosts feeding on different plant species. Three new host records are here reported: *Clemelis massilia* Herting, 1977 developing on *L. botrana* (Denis & Schiffermüller, 1775) living on shoots and inflorescences of *D. gnidium* in Tuscany, *Clausicella suturata* Rondani, 1859 on Ephestia unicolorella subsp. woodiella Richards & Thomson, 1932 and eventually *Neoplectops pomonellae* (Schnabl & Mokrzecki, 1903) on *L. botrana*. The parasitoid-host issue is addressed on three different levels: first we report parasitoid-host records for eleven species of tachinids collected in Italy and Spain on various lepidopteran hosts; then we refer to the parasitoid-host relationships between plants and different lepidopteran species; lastly, due to the great importance of *L. botrana* in viticulture, we provide a preliminary identification key to the puparia of its tachinid parasitoids.

## Materials and methods

Tachinid flies emerged from caterpillars collected in Piedmont, Tuscany, Apulia and Spain on plants belonging to three different plant families: *Quercus* ssp. (*Q. pubescens* and *Q. robur*) (Fagaceae) in Piedmont, grapevine (*Vitis vinifera*, Vitaceae) in Piedmont, Tuscany and Apulia, and *Daphne gnidium* (Thymelaeaceae) in Tuscany and Spain. Puparia were studied by PLS whereas the adult flies, once emerged, were mounted on pins and identified by PC. Moths were mounted on pins and identified by Graziano Bassi, AuL, AnL, and PLS.

Digital images were taken on a Leica Z16 APO stereoscope equipped with a Nikon D5300 digital camera and stacked in a single in-focus image using Helicon Focus 3D (version 3.9.7W) and Zerene Stacker software (version 1.04). All specimens are currently preserved in the collection of the Department of Agriculture, Food and Environment of Pisa University.

**Section A** – Annotated list of the Tachinidae records. The list follows an alphabetical order. Subfamily, tribe, species name, label information, distribution, biological information and puparium description are reported. Additional information may be found under ‘Notes’. Tachinid subfamily, tribe and general distribution are listed accordingly to O’Hara et al. (2019) and Cerretti (2010) for the Italian distribution. Biological and host record information for each species are based on Tschorsnig (2017) and on Cerretti and Tschorsnig (2010). The morphological terminology and characterisation of the tachinid puparia follow Greene (1922), Ziegler (1998) and O’Hara (2005).

**Section B** – Annotated list of records by host plant and Lepidoptera. The list by host plant and Lepidoptera follows an alphabetical order. The lepidopteran species names refer to Fauna Europaea (de Jong et al. 2014).

**Section C** – Preliminary key to the puparia of tachinid flies associated with *L. botrana*. The key is based both on direct observations and on illustrations already available in the literature.

## Results

### A. Annotated list of Tachinid records

#### Subfamily: Exoristinae

##### Tribe: Blondeliini

###### A1. Compsilura concinnata

Taxon classificationAnimaliaTachinidaeTachinidae

(Meigen, 1824)

2DF3A5DF-4C46-5928-95E6-FE7233053AD3

Fig. 1

####### Label information.

Italy, Piedmont: Torino, Santena, oak-hornbeam lowland forest, collected 20.v.1986, emerged 09.vi.1986 ex *Euproctis chrysorrhoea* on *Quercus* sp., P. L. Scaramozzino leg., 1♂, P. Cerretti det.

####### Distribution.

Subcosmopolitan. Italian distribution: north and south Italy, Sicily, Sardinia.

####### Biology.

Parasitoid on a wide range of Lepidoptera and HymenopteraSymphyta. In Italy it has already been reported on E. (Euproctis) chrysorrhoea (Linnaeus, 1758) (Erebidae) in Emilia-Romagna (Faggioli 1937; Cerretti and Tschorsnig 2010) and Sardinia (Delrio and Luciano 1985).

**Figure 1.**
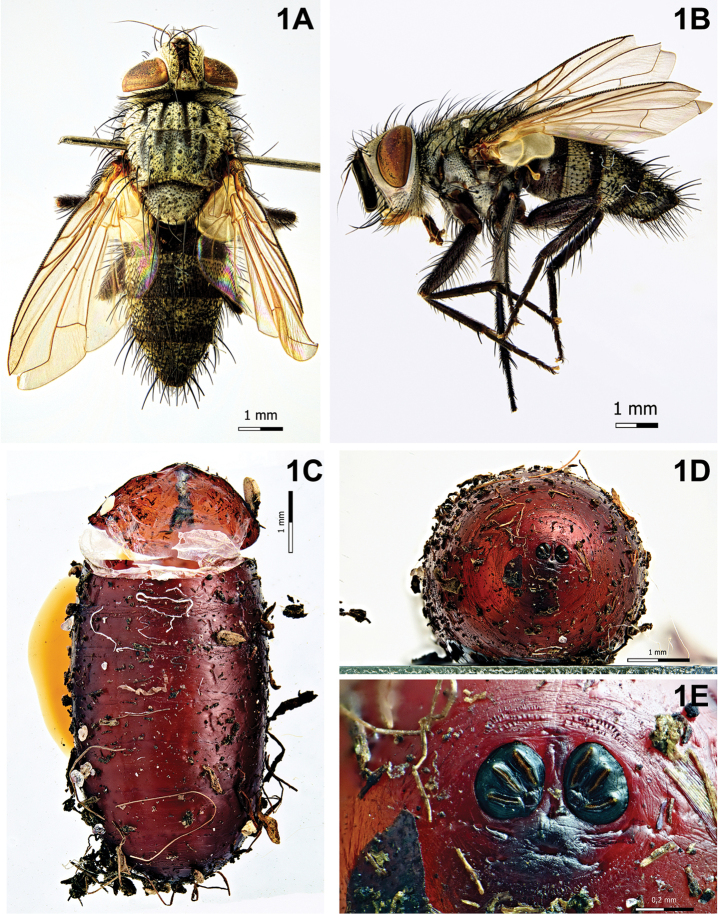
*Compsilura concinnata* (Meigen, 1824). **A** Male, habitus, dorsal view **B** male, habitus, lateral view **C** puparium, dorsal view **D** puparium, posterior end **E** puparium, spiracular plates showing openings.

Puparium (Fig. 1C–E): cylindrical with rounded posterior edges, subshiny, dark red, smooth with circular anterior spinose bands; posterior spiracular plates slightly above level of longitudinal axis and scarcely raised above surface of puparium; each posterior spiracular plate with three linear openings; button round, scarcely defined; anal opening dark, below longitudinal axis at about the same distance of posterior spiracular plates from longitudinal axis.

####### Notes.

In Piedmont three other species of Tachinidae emerged from *E. chrysorrhoea*: *Blondelia nigripes* (Fallén, 1810), *Exorista larvarum* (Linnaeus, 1758) and *Townsendiellomyia nidicola* (Townsend, 1908) (Currado et al. 1988).

##### Tribe: Eryciini

###### A2. Carcelia falenaria

Taxon classificationAnimaliaTachinidaeTachinidae

(Rondani, 1859)

21537BF0-24BC-5C27-A4D9-C26B65FE632B

Fig. 2

####### Label information.

Italy, Piedmont: Torino, Stupinigi, oak-hornbeam lowland forest, 03.vi.1986, ex *Amata* sp. on *Quercus* sp., P. L. Scaramozzino leg., 2♂♂, P. Cerretti det.

####### Distribution.

Palaearctic. Italian distribution: north and south Italy, Sicily.

####### Biology.

Parasitoid on LepidopteraErebidae of the genus *Amata* Fabricius, 1807. In Italy it has been obtained in Veneto on *A. kruegeri* (Ragusa, 1904) and in Sicily on *Amata* sp. (Cerretti and Tschorsnig 2010).

####### Notes.

Two adults emerged from the same host larva; the larvae pupated within the body of the host larva and the adults emerged from cut-like openings made on the host exoskeleton.

**Figure 2.**
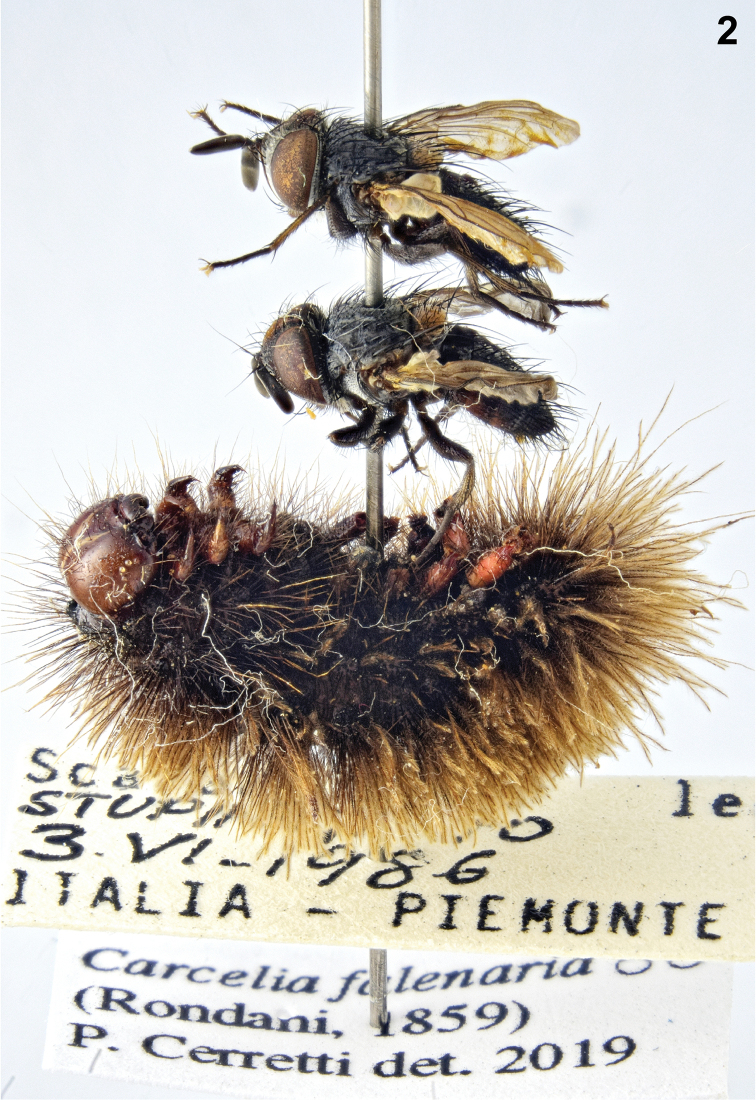
*Carcelia falenaria* (Rondani, 1859), two males emerged from *Amata* sp. larva.

###### A3. Phryxe cf. nemea

Taxon classificationAnimaliaTachinidaeTachinidae

(Meigen, 1824)

DDA01DAC-D235-5604-92AA-86D7D6B86565

####### Label information.

Italy, Piedmont: Torino, Venaria, La Mandria, oak-hornbeam lowland forest, vi.1988, ex *Tortrix viridana* on *Quercus robur*, P. L. Scaramozzino leg., 1♂, P. Cerretti det. The specimen emerged from the cocoon with wings still partially folded.

####### Distribution.

Palaearctic. Italian distribution: north and south Italy, Sardinia.

####### Biology.

Parasitoid on a wide range of Lepidoptera and seldom HymenopteraSymphyta. In Italy it has already been obtained from *T. viridana* Linnaeus, 1758 (Tortricidae) on *Q. robur* in Sardinia (Delrio et al. 1988).

**Figure 3.**
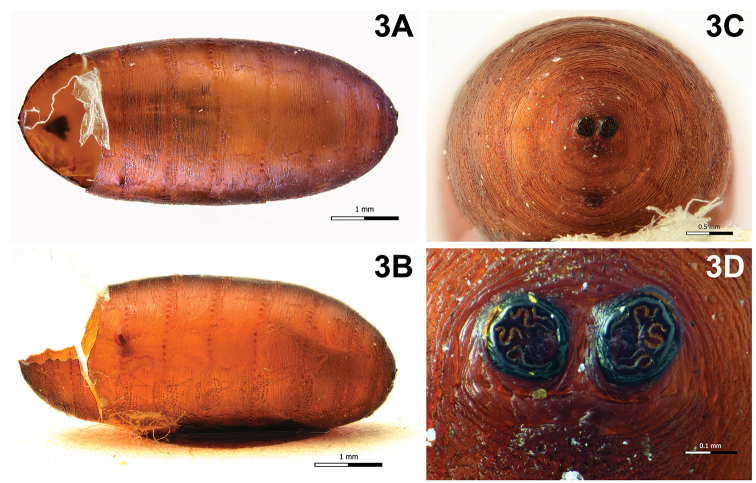
Phryxe cf. nemea (Meigen, 1824). **A** Puparium, dorsal view **B** puparium, lateral view **C** puparium, posterior end **D** puparium, spiracular plates showing openings.

Puparium (Fig. 3A–D): cylindrical with rounded posterior edges, dull, light brown, surface transversally striated, with circular anterior spinose bands; posterior spiracular plates on longitudinal axis and scarcely raised above surface of puparium; each posterior spiracular plate with four serpentine openings; button round and large; anal opening concolourous, below longitudinal axis, located at some distance from posterior spiracular plates.

###### A4. Pseudoperichaeta nigrolineata

Taxon classificationAnimaliaTachinidaeTachinidae

(Walker, 1853) – First record on C. pronubana in Italy

9ACCD5C6-7966-5CE7-B563-9E911A9BE1B8

Fig. 4

####### Label information.

Italy, Tuscany: Pisa, P. N. San Rossore, coastal mixed forest of stone pine, maritime pine and holm oak, 29.viii.2017, ex *Cacoecimorpha pronubana* on *Daphne gnidium*, A. Loni & P. L. Scaramozzino leg., 1♂, P. Cerretti det.

####### Distribution.

Palaearctic and Oriental. Italian distribution: north and south Italy, Sicily, Sardinia.

####### Biology.

Parasitoid on several lepidopteran families. It has been reared from *C. pronubana* (Hübner, [1799]) (Tortricidae) in France (IOBC-List 2 1957), Ukraine (Richter 1996) and United Kingdom (Collin 1909; Ford et al. 2000). This is the first record for this species on *C. pronubana* in Italy. This species is also recorded as parasitoid of *L. botrana* (Tab. 2).

**Figure 4.**
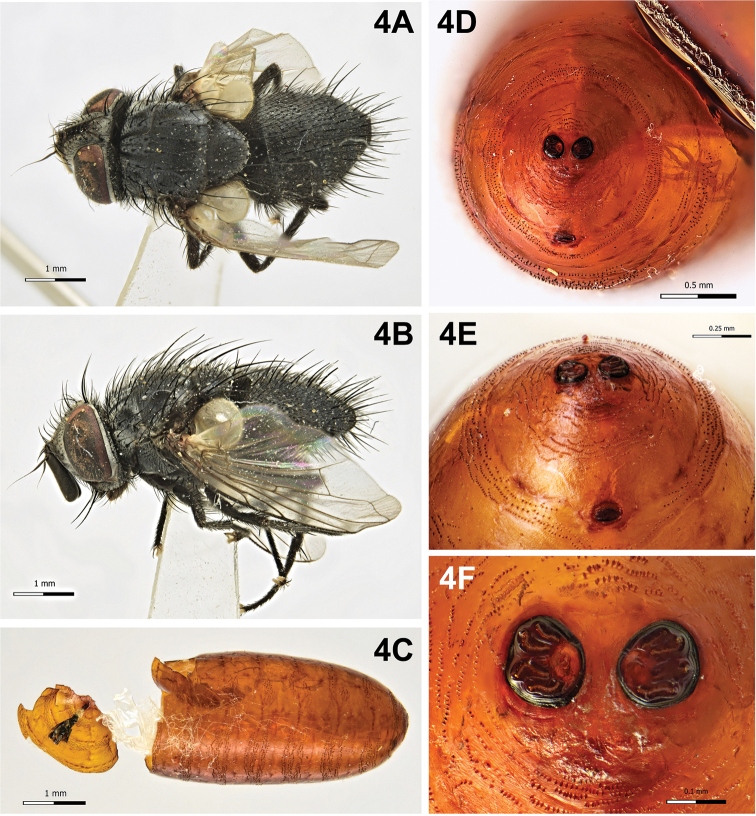
*Pseudoperichaeta nigrolineata* (Walker, 1853). **A** Male, habitus, dorsal view **B** male, habitus, lateral view **C** puparium, lateral view, anterior end detached **D** puparium, posterior end **E** puparium, posterior end, ventral view, showing anal opening **F** puparium, spiracular plates showing openings.

Puparium (Fig. 4C–F): cylindrical with rounded posterior edges, subshining, light yellowish-brown, smooth with circular anterior spinose bands; posterior spiracular plates slightly above longitudinal axis and on surface of puparium; each posterior spiracular plate with four linear or slightly curved openings; button round and large; anal opening dark, below longitudinal axis, located at some distance from posterior spiracular plates.

##### Tribe: Exoristini

###### A5. Bessa parallela

Taxon classificationAnimaliaTachinidaeTachinidae

(Meigen, 1824) – First record on T. viridiana in Italy

035E2CE3-E033-5497-86E9-DBEBAEF8E835

Fig. 5

####### Label information.

Italy, Piedmont: Torino, Grange di Brione, mixed oak forest, 17.v.1990, ex *Tortrix viridana* on *Quercus* sp., P. L. Scaramozzino leg., 1♀, P. Cerretti det.

####### Distribution.

Palaearctic and Oriental. Italian distribution: north and south Italy.

####### Biology.

Parasitoid mainly on Lepidoptera, with Coleoptera or HymenopteraSymphyta as unusual hosts. It has already been recorded on *T. viridana* in several regions of North, Central and East Europe. This is the first record for this species on *T. viridana* in Italy. This species is also recorded for *L. botrana* (Tab. 2), even if the single record in literature (Jordan 1915) is from specimens obtained from lab parasitisation tests (Tschorsnig 2017).

**Figure 5.**
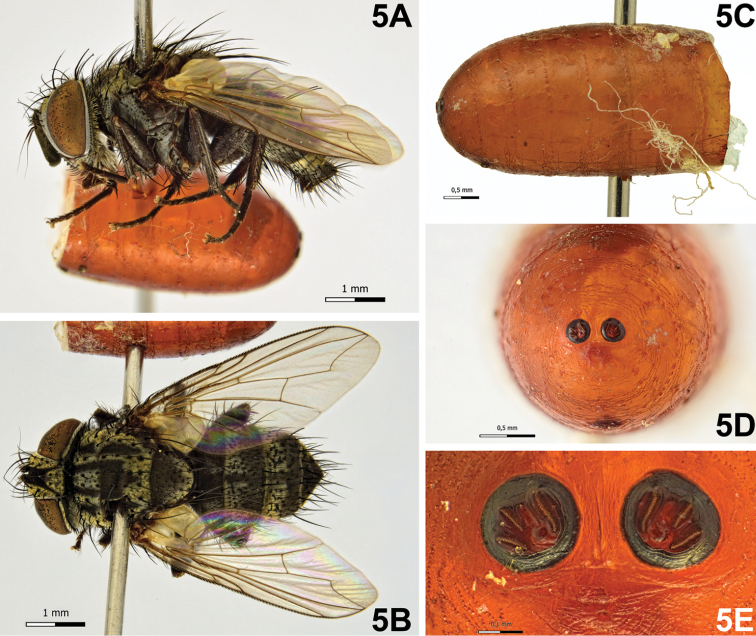
*Bessa parallela* (Meigen, 1824). **A** Female, habitus, lateral view **B** female, habitus, dorsal view **C** puparium, lateral view **D** puparium, posterior end **E** puparium, spiracular plates showing openings.

Puparium (Fig. 5C–E): cylindrical with rounded posterior edges, subshiny, yellowish-brown, smooth with circular anterior spinose bands; posterior spiracular plates slightly above level of longitudinal axis and scarcely raised above surface of puparium; each posterior spiracular plate with three linear openings; button round, scarcely defined; anal opening dark, below longitudinal axis and very remote from posterior spiracular plates on ventral surface.

##### Tribe: Goniini

###### A6. Clemelis massilia

Taxon classificationAnimaliaTachinidaeTachinidae

Herting, 1977 – First host-record

105E9C72-FD60-5A18-871F-6BD65A2C4AB2

Fig. 6

####### Label information.

Italy, Tuscany: Pisa, P. N. San Rossore, coastal mixed forest of stone pine, maritime pine and holm oak, 28.v.2015, ex *Lobesia botrana* on *Daphne gnidium*, A. Loni & P. L. Scaramozzino leg., 1♂, P. Cerretti det.

####### Distribution.

Palaearctic. Italian distribution: north and south Italy.

####### Biology.

This is the first known host record for *C. massilia*. A similar and more common species, *C. pullata* (Meigen, 1824), has been obtained from several families of Lepidoptera, including Tortricidae as *Archips podana* (Scopoli, 1763), *A. rosana* (Linnaeus, 1758), *Choristoneura diversana* (Hübner, [1814–1817]) and *Pandemis heparana* (Denis & Schiffermüller, 1775).

**Figure 6.**
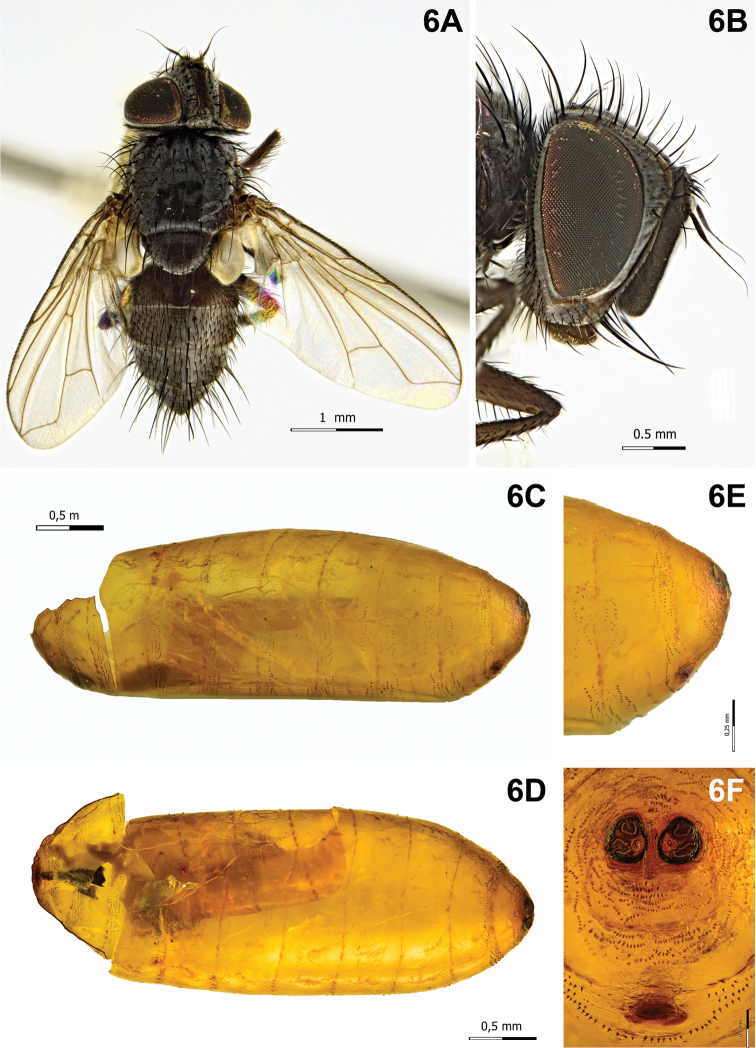
*Clemelis massilia* Herting, 1977. **A** Male, habitus, dorsal view **B** male, head, lateral view **C** puparium, lateral view **D** puparium, dorsal view **E** puparium, posterior end, lateral view **F** puparium, anal opening and spiracular plates showing openings.

Puparium (Fig. 6C–F): sub-cylindrical with posterior edge slightly depressed dorsally and broadly rounded ventrally, subshiny, yellowish-brown, smooth with incomplete, anterior spinose circular bands; posterior spiracular plates clearly above longitudinal axis and on surface of puparium; each posterior spiracular plate with three linear or curved openings; button round, scarcely defined; anal opening dark, below longitudinal axis and remote from posterior spiracular plates, half the distance from ventral surface.

####### Notes.

The adult we obtained emerged from a puparium inside the host cocoon together with the remains of a mature larva of *L. botrana*.

##### Tribe: Winthemiini

###### A7. Nemorilla maculosa

Taxon classificationAnimaliaTachinidaeTachinidae

(Meigen, 1824) – First record on L. botrana in Italy

2C406696-21FB-55C3-80E1-A007BAD8CAA5

Fig. 7

####### Label information.

Italy, Tuscany: Pisa, P. N. San Rossore, coastal mixed forest of stone pine, maritime pine and holm oak, 24.ix.2015 ex *Lobesia botrana* on *Daphne gnidium*, A. Loni & P. L. Scaramozzino leg., 1 larva with two macrotipic eggs; 01.x.2015, same data, 1 specimen (sex not determinable), P. Cerretti det.; same data, 07.vi.2017, 1 specimen (sex not determinable); same data, 29.viii.2017, 1 puparium; same data, 14.ix.2017, 1 specimen (sex not determinable); same data, 07.vi.2018, 1♀.

####### Distribution.

Palaearctic and Oriental. Italian distribution: north and south Italy, Sicily, Sardinia.

####### Biology.

Parasitoid of a wide range of lepidopteran families. It has already been obtained from *L. botrana* in Bulgaria (Trenchev 1980), Iran (Shoukat 2012), Spain (Coscollá 1981) and Ukraine (Telenga 1934). This is the first record for this species on *L. botrana* in Italy. In Morocco, *N. maculosa* was found on *Cryptoblabes gnidiella* (Millière, 1867) (Pyralidae), which often cohabits the same nests built by *L. botrana* on *D. gnidium* (Scaramozzino et al. 2017b). The biology and preimaginal stages of *N. maculosa* have been studied and illustrated in detail by Mellini (1964).

**Figure 7.**
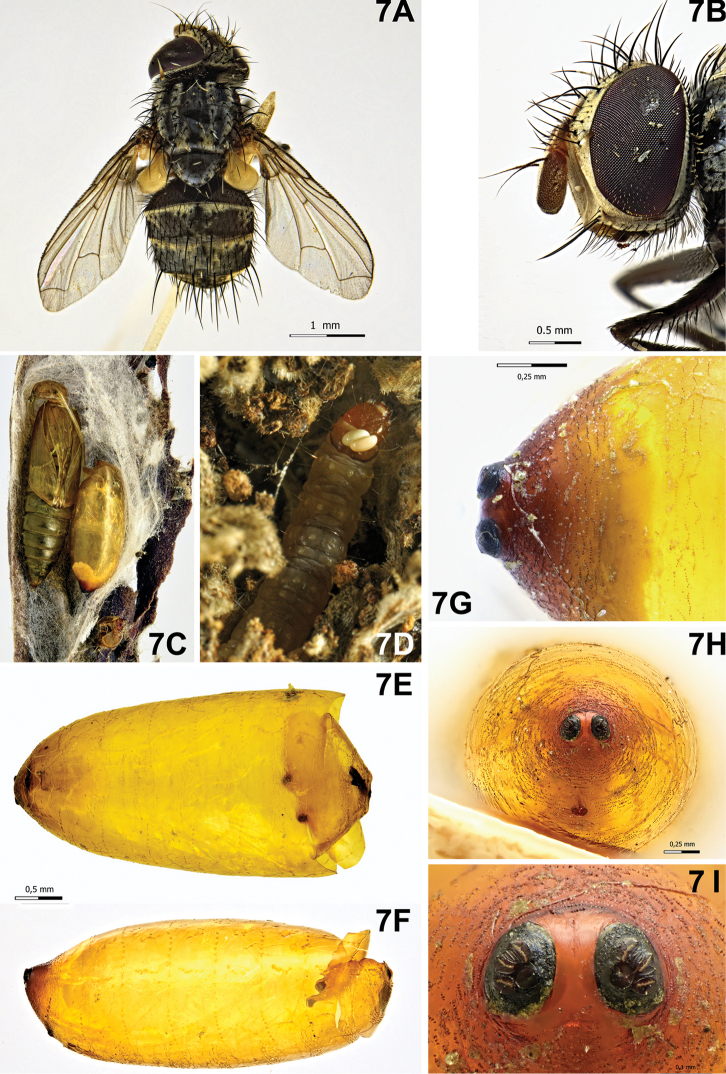
*Nemorilla maculosa* (Meigen, 1824). **A** Female, habitus, dorsal view **B** female, head, lateral view **C** puparium next to remains of *Lobesia botrana* chrysalis on *Daphne gnidium***D** larva of *L. botrana* with two macrotipic eggs **E** puparium, ventral view **F** puparium, lateral view **G** puparium, posterior end, dorsal view **H** puparium, posterior end, posterior view **I** puparium, spiracular plates showing openings.

Puparium (Fig. 7E–I): sub-cylindrical with posterior edge slightly depressed dorsally and broadly rounded ventrally, shining, yellow, smooth with not well defined circular anterior spinose bands; posterior spiracular plates clearly above longitudinal axis and slightly raised above surface of puparium; each posterior spiracular plate with three small linear openings and with some scars in between; button round and large, defined; anal opening red, below longitudinal axis and remote from posterior spiracular plates, half the distance from ventral surface.

####### Notes.

According to Tschorsnig (2017), records of *Nemorilla floralis* (Fallén, 1810) on *L. botrana* (Telenga 1934, Trenchev 1980, Coscollá 1981) are probably misidentifications for *N. maculosa*. The puparium of this tachinid was found inside the cocoon of *L. botrana*, next to the remains of the chrysalis or the mature larva (Fig. 7C). Moreover, during our observations, we found the eggs of a tachinid (Fig. 7D) on the pronotum of two mature EGVM larvae. The first larva only had one egg from which emerged a malformed and unidentifiable tachinid fly, though the remains of its puparium were very similar to those of *N. maculosa*. The second larva bore two tachinid eggs but it unfortunately died before parasitoid emergence. This species was obtained from *L. botrana* in two different periods of the year: in June, and from the last days of August to the end of September.

#### Subfamily: Tachininae

##### Tribe: Graphogastrini

###### A8. Phytomyptera nigrina

Taxon classificationAnimaliaTachinidaeTachinidae

(Meigen, 1824)

E56DA4BA-BA63-5672-B33E-47FE4C8D19D7

####### Label information.

Italy, Piedmont: Cuneo, Barbaresco, vineyard, 31.v.2018, ex *Lobesia botrana* on *Vitis vinifera*, R. Ricciardi leg., 1♂ 2♀♀, P. Cerretti det. Italy, Tuscany: Livorno, Castagneto Carducci, vineyard, 14.vi.2005, ex *Lobesia botrana* on *Vitis vinifera*, 1♂, P. Cerretti det.; Pisa, Cerreto Guidi, vineyard, 20.vi.2005, ex *Lobesia botrana* on *Vitis vinifera*, 1♀, P. Cerretti det.; same data, 23.vi.2005, 1♀; same data, 28.vi.2005, 1♀; same data, 29.vi.2005, 1♀; same data, 29.vii.2005, 1♂; Pisa, Terricciola, vineyard, 10.viii.2005, ex *Lobesia botrana* on *Vitis vinifera*, 1♀, P. Cerretti det.; Pisa, P. N. San Rossore, coastal mixed forest of stone pine, maritime pine and holm oak, 31.viii.2014, ex *Lobesia botrana* on *Daphne gnidium*, A. Loni & P. L. Scaramozzino leg., 1♀, P. Cerretti det.; same data, 07.vi.2017, 1♀. Italy, Apulia: Brindisi, Masseria Maime, vineyard, 15.v.2018, ex *Lobesia botrana* on *Vitis vinifera*, R. Ricciardi leg., 1♂, P. Cerretti det. Spain: Girona, Port de la Selva, overgrown vineyard, 25.viii.2014, ex *Lobesia botrana* on *Daphne gnidium*, M. Generani & P. L. Scaramozzino leg., 3♂♂ 7♀♀, P. Cerretti det.; Girona, Llança, Serra de Carbet, overgrown vineyard, 21.viii.2014, ex *Lobesia botrana* on *Daphne gnidium*, M. Generani & P. L. Scaramozzino leg., 1♀, P. Cerretti det.

####### Distribution.

Palaearctic. Italian distribution: north and south Italy, Sicily, Sardinia.

####### Biology.

Parasitoid on about 30 hosts belonging to different lepidopteran families. In Italy, it is a renowned *L. botrana* parasitoid (Scaramozzino et al. 2017a) and it is considered one of the main control agents of *L. botrana* in the vineyards, where it can significantly contribute in reducing the summer population of the moth (Bagnoli and Lucchi 2006; Thiéry et al. 2006); it has been obtained from *L. botrana* in vineyards in Piedmont (Colombera et al. 2001), Trentino (Catoni 1914), Veneto (Marchesini and Dalla Montà 1992; 1994), Tuscany (Bagnoli and Lucchi 2006), Campania (Silvestri 1912), Calabria (Laccone 2007) and Apulia (Laccone 1978) and from *L. botrana* nests on *D. gnidium* in Apulia (Nuzzaci and Triggiani 1982) and Sardinia (Luciano et al. 1988). In Spain, it has been reported from *L. botrana* in vineyards by Coscollá (1981). It also parasitises *Eupoecilia ambiguella* (Hübner, 1796) (Tortricidae), another important pest of the grapevine. The biology and preimaginal stages of *P. nigrina* have been studied and illustrated in detail by Mellini (1954), and the life-history was briefly reviewed by Andersen (1988).

**Figure 8.**
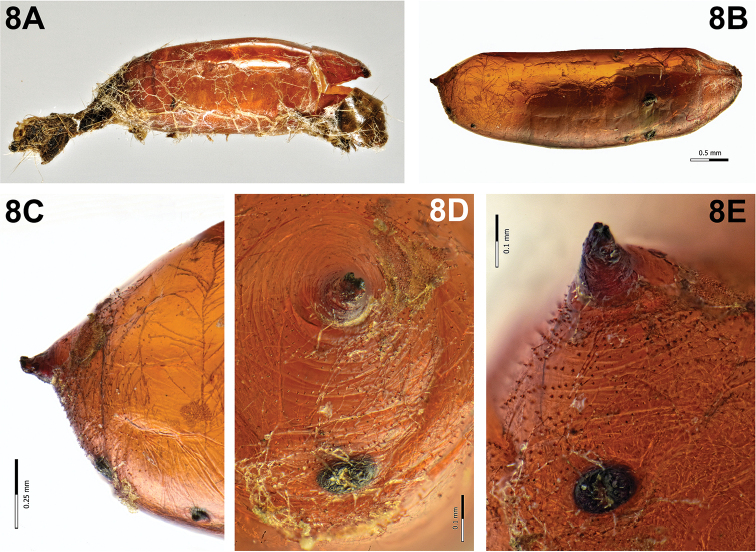
*Phytomyptera nigrina* (Meigen, 1824). **A** Puparium covered with the host larva skin remains **B** puparium, lateral view **C** puparium, posterior end, lateral view **D** puparium, anal opening and reduced spiracular plates **E** puparium, anal opening and spiracular plates reduced and borne on a subconical projection.

Puparium (Fig. 8A–E): sub-cylindrical with both edges slightly depressed dorsally and broadly rounded ventrally, shining, red-brown, smooth with scarce spines towards edges; posterior spiracular plates slightly above level of longitudinal axis, borne on a subconical projection; posterior spiracular plate reduced, without openings; anal opening round and dark, below longitudinal axis and remote from posterior spiracular plates, half distance from ventral surface. It was covered with remains of host larva cuticle.

####### Notes.

Only one specimen of *P. nigrina* was obtained from EGVM larvae during a 4-year survey on *D. gnidium* in San Rossore Natural Reserve (Tuscany). In this context, *Actia pilipennis* resulted instead the most abundant species of Tachinidae parasitising EGVM. Contrariwise, in other researches on the same plant, it was definitely the most common species among the parasitoids of *L. botrana* (Nuzzaci and Triggiani 1982, Luciano et al. 1988); in Apulia it attacked 30% of the larvae, in Sardinia it was the most common parasitoid on spurge flax while it was completely absent on the vine. In our occasional samplings on *D. gnidium* in the north of Spain (Girona, Catalonia) during the summer, *P. nigrina* was the only parasitoid obtained from EGVM in summer.

##### Tribe: Leskiini

###### A9. Clausicella suturata

Taxon classificationAnimaliaTachinidaeTachinidae

Rondani, 1859 – New record on Ephestia unicolorella subsp. woodiella Richards & Thomson

BC0C6D61-1F23-5AAA-AEFF-F9B09FBFC4E4

Fig. 9

####### Label information.

Italy, Tuscany: Pisa, Terricciola, vineyard, 08.iii.2006, emerged 20.iii.2006, ex Ephestia unicolorella subsp. woodiella on *Vitis vinifera* (bark), A. Lucchi leg., 6♂♂ 8♀♀, P. Cerretti det.

####### Distribution.

Palaearctic. Italian distribution: north and south Italy, Sicily, Sardinia.

####### Biology.

Parasitoid on LepidopteraPyralidae. So far, it has been obtained from *Apomyelois ceratoniae* (Zeller, 1839), *Cadra figulilella* (Gregson, 1871) and *Euzophera bigella* (Zeller, 1848); the latter represents the only Italian host record for this species (Reggiani et al. 2006). This is the first record for this species on E. unicolorella subsp. woodiella Richards & Thomson, 1932 (Pyralidae).

**Figure 9.**
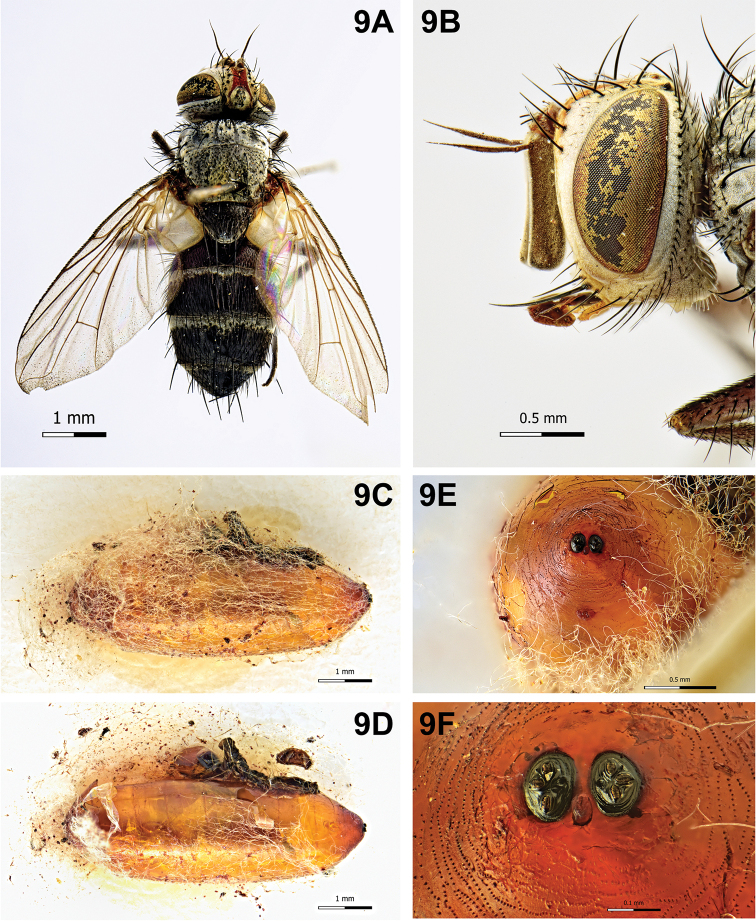
*Clausicella suturata* Rondani, 1859. **A** Male, habitus, dorsal view **B** male, head, lateral view **C** puparium, covered with host silk **D** puparium, lateral view **E** puparium, posterior end **F** puparium, spiracular plates showing openings.

Puparium (Fig. 9C–F): sub-cylindrical with posterior edge slightly depressed dorsally and broadly rounded ventrally, shining, yellow, smooth with scarce spines towards edges; posterior spiracular plates shining, on longitudinal axis and raised above surface of puparium; each posterior spiracular plate with three small linear openings on a broad defined ridge; button round and small, defined; anal opening red, below the longitudinal axis and remote from posterior spiracular plates, half distance from ventral surface.

##### Tribe: Neaerini

###### A10. Neoplectops pomonellae

Taxon classificationAnimaliaTachinidaeTachinidae

(Schnabl & Mokrzecki, 1903) – New record on L. botrana

316D9142-620D-5D41-B01D-2D32306B5B10

Fig. 10

####### Label information.

Italy, Tuscany: Pisa, P. N. San Rossore, coastal mixed forest of stone pine, maritime pine and holm oak, 11.ix.2013, emerged 07.x.2013 from *Lobesia botrana* nests in cages collected on *Daphne gnidium*, A. Loni & P. L. Scaramozzino leg., 2♂♂, P. Cerretti det.

####### Distribution.

Palaearctic. Italian distribution: south Italy.

####### Biology.

Parasitoid on LepidopteraTortricidae, particularly on *Cydia pomonella* (Linnaeus, 1758). The only known Italian host record for this species is on *Gypsonoma* sp. Meyrick, 1895 (Ford and Shaw 1991). This is the first record for this species on *L. botrana* (Tortricidae).

####### Notes.

Two males of *N. pomonellae* were obtained during the initial trial of our research in San Rossore. Numerous EVGM nests were put together in a cage aiming to a rough estimate of the parasitoid that could be obtained but neither puparia nor adults were found again.

**Figure 10.**
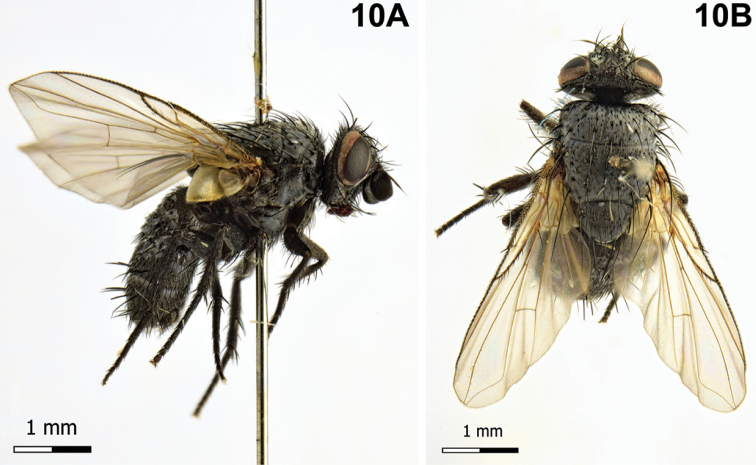
*Neoplectops pomonellae* (Schnabl & Mokrzecki, 1903). **A** Male, habitus, lateral view **B** male, habitus, dorsal view.

##### Tribe: Siphonini

###### A11. Actia pilipennis

Taxon classificationAnimaliaTachinidaeTachinidae

(Fallén, 1810)

5BB4616F-DBC8-5913-BA11-A196D000EB98

####### Label information.

Italy, Piedmont: Alessandria, Sacro Monte di Crea, mixed oak forest, 19.v.1988, ex *Tortrix viridiana* on *Quercus pubescens*, P. L. Scaramozzino leg., 4♀♀, P. Cerretti det.; Torino, Brione, Monte Musiné, mixed oak forest, 31.v.1987, ex *Tortrix viridiana* on *Quercus pubescens*, P. L. Scaramozzino leg., 1♀, P. Cerretti det.; same data, 04.vi.1988, 1♂; Torino, Cavagnolo, mixed oak forest, 11.v.1988, ex *Tortrix viridiana* on *Quercus pubescens*, P. L. Scaramozzino leg., 3♂♂ 2♀♀, P. Cerretti det.; Torino, Stupinigi, oak-hornbeam lowland forest, 26.v.1986, ex *Tortrix viridiana* on *Quercus robur*, P. L. Scaramozzino leg., 1♀, P. Cerretti det.; same data, 03.vi.1986, 1♀; same data, 10.v.1988, 1♀; same data, 15.v.1988, 4♂♂ 2♀♀; same data, 16.v.1988, 1♂. // Italy, Tuscany: Pisa, P. N. San Rossore, coastal mixed forest of stone pine, maritime pine and holm oak, 10.vi.2012, ex *Lobesia botrana* on *Daphne gnidium*, A. Loni & P. L. Scaramozzino leg., 3♂♂ 1♀, P. Cerretti det.; same data, 15.vii.2012, 1♂; same data, 11.ix.2013, 1♀; same data, 29.v.2014, 2♀♀; same data, 15.vii.2014, 1♀; same data, 31.vii.2014, 1♂; same data, 11.vi.2015, 1♂; same data, 09.v.2017, 1♀; same data, 24.v.2017, 1♂ 1♀; same data, 30.v.2017, 2♀♀; further males and females emerged in cages from *Lobesia botrana* nests, collected on *D. gnidium*: same data, 27.vi.2014, 2♂♂; same data, 07.vi.2017, 1♂ 1♀; same data, 28.vi.2017, 1♂ 1♀.

####### Distribution.

Palaearctic and Oriental. Italian distribution: north and south Italy, Sicily, Sardinia.

####### Biology.

Parasitoid mainly on Tortricidae. It has already been recorded on *T. viridana* in several Palaearctic countries, including Italy (Silvestri 1923). In French vineyards, it has been reported by Martinez (2012) on the tortricid *Sparganothis pilleriana* (Denis & Schiffermüller, 1775) and more recently by Delbac et al. (2015) on *L. botrana*. In Italy, Scaramozzino et al. (2017a) recorded *Actia pilipennis* obtained from *L. botrana* feeding on *D. gnidium*.

**Figure 11.**
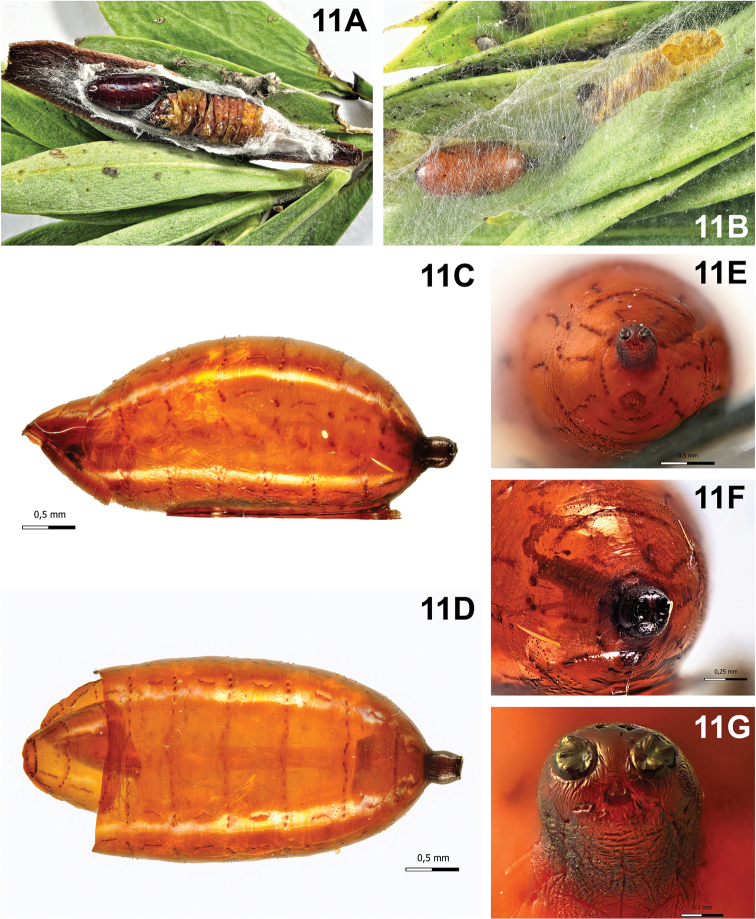
*Actia pilipennis* (Fallén, 1810). **A** Puparium inside *Lobesia botrana* cocoon, next to host larva remains **B** puparium next to *L. botrana* mature larva remains **C** puparium, lateral view **D** puparium, dorsal view **E** puparium, posterior end showing anal opening and spiracular plates **F** puparium, posterior end **G** puparium, spiracular plates borne on a cylindrical projection, showing openings.

Puparium (Fig. 11C–G): suboval with rounded posterior edges, shining, orange-yellow, smooth with incomplete anterior bands of spines; posterior spiracular plates on the longitudinal axis and borne on a cylindrical projection; posterior spiracular plate small, with three small linear openings; button round and small, defined; anal opening concolourous, just below the longitudinal axis.

####### Notes.

In Piedmont, from 1986 to 1988 *A. pilipennis* was the tachinid most frequently attacking *T. viridana* larvae on oaks and it was found inside the host cocoons in the rolled leaves. In San Rossore, it resulted the most abundant tachinid parasitoid of *L. botrana* on *D. gnidium*, as above mentioned. Normally its puparia are found inside the cocoon of the EGVM, near the remains of the host larva (Fig. 11A, B), and only rarely the fly emerges from the mature larva that has not yet woven its cocoon and pupate between the leaves of its nest.

### B. Annotated list of records by host plant and Lepidoptera

#### Species on *Quercus* spp. [Fagales, Fagaceae]

In Europe, as well as throughout the northern hemisphere, oak is an important component of deciduous forests, representing an extremely species-rich tree. In Britain, Southwood (1961) reports 284 insect species associated with oak, most of them belonging to the order Lepidoptera and Coleoptera (237 species) (Morris 1974). For Western Palaearctic, Soria (1988) lists 453 species of foliage-feeding Lepidoptera, belonging to 37 families. Not all insect species found on oak trees are primarily associated with these plants though, and even fewer are those who can cause considerable damage. Indeed, Klimetzek (1993) reports 136 insect pests associated with oak in Europe. Both the brown tail moth (*Euproctis chrysorrhoea*) and the green oak leaf-roller (*Tortrix viridana*) are considered two of the main pests of oak in Europe (Day and Leather 1997).

##### B1. Euproctis (Euproctis) chrysorrhoea (Linnaeus, 1758) (Lepidoptera, Erebidae, Lymantriinae)

Forty species of Tachinids are reported on *E. chrysorrhoea*: only 27 are certain, whereas the remaining are either dubious or incorrect (Tschorsnig 2017). In Italy, ten species have been reported (Cerretti and Tschorsnig 2010).

Associated parasitoid:

***Compsilura concinnata* (Meigen, 1824**) [A1]

##### B2. *Tortrix viridana* Linnaeus, 1758 (Lepidoptera, Tortricidae, Tortricinae)

Thirty-four Tachinidae are reported on *T. viridana*: only 22 of them are certain, the remaining are either dubious or incorrect (Tschorsnig 2017). In Italy, eleven species have been reported (Cerretti and Tschorsnig 2010).

Associated parasitoids:

***Actia pilipennis* (Fallén, 1810)** [A11]

***Bessa parallela* (Meigen, 1824)** [A5]

**Phryxe cf. nemea (Meigen, 1824)** [A3]

#### Species on *Daphne gnidium* Linnaeus, 1753 [Malvales, Thymelaeaceae]

Numerous lepidopteran species coexist on the spurge flax, which is considered as the EGVM wild host plant (Nuzzaci and Triggiani 1982, Luciano et al. 1988, Scaramozzino et al. 2017b). In the Natural Reserve of San Rossore, we commonly found the following species on *D. gnidium*: *L. botrana*, *Cacoecimorpha pronubana* (Lepidoptera, Tortricidae), *Anchinia cristalis* (Scopoli, 1763) (Lepidoptera, Elachistidae), *Phyllobrostis fregenella* Hartig, 1941 (Lepidoptera, Lyonetiidae), *Cryptoblabes gnidiella* (Lepidoptera, Pyralidae), and *Gymnoscelis rufifasciata* (Haworth, 1809) (Lepidoptera, Geometridae). *L. botrana*, *C. pronubana* and *A. cristalis* are typically “leaf rollers”, i.e., their larvae form a shelter (nest) by rolling up the leaves on which they live; *P. fregenella* is a leaf miner, while *C. gnidiella* and *G. rufifasciata* are commonly found inside the nests of *L. botrana*. *Nemorilla maculosa* [A7], one of the Tachinidae obtained in San Rossore from *L. botrana*, is also mentioned as emerged from *C. gnidiella* (Tschorsnig 2017). *Pseudoperichaeta nigrolineata*, also reported from *L. botrana*, have been found in a single specimen on *C. pronubana*.

##### B3. *Cacoecimorpha pronubana* (Hübner, [1799]) (Lepidoptera, Tortricidae, Tortricinae)

Eight species of Tachinidae are reported on *C. pronubana*, two of which have been found in Italy (Cerretti and Tschorsnig 2010, Tschorsnig 2017).

Associated parasitoid:

***Pseudoperichaeta nigrolineata* (Walker, 1853)** [A4]

##### B4. *Lobesia botrana* (Denis & Schiffermüller, 1775) (Lepidoptera, Tortricidae, Olethreutinae)

See Discussion.

Associated parasitoids:

***Actia pilipennis* (Fallén, 1810)** [A11]

***Clemelis massilia* Herting, 1977** [A6]

***Nemorilla maculosa* (Meigen, 1824)** [A7]

***Neoplectops pomonellae* (Schnabl & Mokrzecki, 1903)** [A10]

***Phytomyptera nigrina* (Meigen, 1824)** [A8]

#### Species on *Vitis vinifera* Linnaeus, 1753 [Vitales, Vitaceae]

##### B5. Ephestia unicolorella subsp. woodiella Richards & Thomson, 1932 (Lepidoptera, Pyralidae)

Larvae of this species can be found inside bunches of grapes and feed on the dried berries. They hibernate as mature larvae in the cocoon, on the woody parts of the vine or on the support poles. So far, no tachinids have been found on this species (Tschorsnig 2017).

Associated parasitoid:

***Clausicella suturata* Rondani, 1859** [A9]

##### B4. *Lobesia botrana* (Denis & Schiffermüller, 1775) (Lepidoptera, Tortricidae, Olethreutinae)

See Discussion.

Associated parasitoid:

***Phytomyptera nigrina* (Meigen, 1824)** [A8]

#### Species with unidentified host plant

##### B6. *Amata* sp. Fabricius, 1807 (Lepidoptera, Erebidae, Arctiinae)

Two *Amata* spp. are present in Piedmont: *A. marjana* (Stauder, 1913) [= Amata (Syntomis) kruegeri (Ragusa, 1904)] and *A. phegea* (Linnaeus, 1758) (Bassi pers. comm.). *Amata marjana* feeds on Dipsacaceae (*Knautia* spp.), Asteraceae (*Centaurea* spp., *Artemisia* and *Achillea* spp.) or Fabaceae (*Oxytropis* and *Anthyllis* spp.) (de Freina 2008), whereas *A. phegea* feeds on Graminee (Robinson et al. 2010). Five tachinid species are known to parasitise *Amata* spp. with *Carcelia falenaria* being the most frequently mentioned (Tschorsnig 2017).

Associated parasitoid:

***Carcelia falenaria* (Rondani, 1859)** [A2]

### C. Preliminary key to the puparia of tachinid flies associated with *Lobesia botrana*

The present key includes a strict selection of species, mainly based on the ones directly raised for this study. Puparia of *Neoplectops pomonellae* are unknown; description of the puparium of *Elodia morio* is based on Zuska (1963).

**Table:** 

1	Posterior spiracular plates rising on a median projection (Figs 8C, 11F)	**2**
–	Posterior spiracular plates not rising on a median projection (Figs 4E, 6E, 7G)	**4**
2	Posterior spiracular plates borne upon two separate projections making the posterior end of median projection distinctly bifid (see Zuska 1963: fig. 41). Posterior spiracular openings fused into a C-shaped pseudoslit (see Zuska 1963: fig. 55)	***Elodia morio***
–	Posterior spiracular plates not borne on two separate projections, so the posterior end of median projection not bifid (Figs 8C, 11F)	**3**
3	Posterior spiracular plates with tree linear openings (Fig. 11G). Posteromedian projection subcylindrical (Fig. 11F). Puparium suboval in shape (Fig. 11C). Pupariation taking place within the silky cocoon but outside the host’s remains; puparium not covered by the cuticle of the host	***Actia pilipennis***
–	Posterior spiracular plates very small and openings not clearly visible. Posteromedian projection subconical (Fig. 8C–E). Puparium subcylindrical in shape (Fig. 8B). Pupariation usually taking place within host’s remains so that puparium is covered by the cuticle of the host larva (Fig. 8A)	***Phytomyptera nigrina***
4	Posterior spiracular plates with four either linear or curved openings (Figs 4F, 12C). Posterior end of puparium, in lateral view, almost hemispherical, i.e., posterodorsal and posteroventral portions of puparium (with respect to posterior spiracles) roundly convex (Figs 4C, 12A)	**5**
–	Posterior spiracular plates with tree either linear or sinuous openings (Figs 5E, 6F, 7I). Posterior end of puparium, in lateral view, with posterodorsal and posteroventral portions roundly convex (Fig. 5C) or with posterodorsal portion depressed and ventral portion broadly convex (Figs 6C, 7F)	**6**
5	Space between the two posterior spiracular plates as long as the diameter of a spiracular plate (Fig. 12C). Spiracular plates with small button (Fig. 12C)	***Eurysthaea scutellaris***
–	Space between the two posterior spiracular plates long less than half the diameter of a spiracular plate (Fig. 4F). Spiracular plates with large button (Fig. 4F)	***Pseudoperichaeta nigrolineata***
6	Posterior end of puparium, in lateral view, almost hemispherical, i.e., posterodorsal and posteroventral portions of puparium (with respect to posterior spiracles) roundly convex (Fig. 5C); spiracular plates arising at about level of midline of puparium in lateral view. Posterior spiracular plate with tree linear openings (Fig. 5E)	***Bessa parallela***
–	Posterior end of puparium, in lateral view, not hemispherical, i.e., with posterodorsal portion slightly depressed anterior to posterior spiracular plate and ventral portion broadly convex; spiracular plates arising high above midline of puparium in lateral view (Figs 6C, 7F). Posterior spiracular plate with tree sinuous (Fig. 6F) or linear openings (Fig. 7I)	**7**
7	Posterior spiracular plates flat, lying on surface of puparium, with tree sinuous openings (Fig. 6F)	***Clemelis massilia***
–	Posterior spiracular plates slightly raised above surface of puparium, with tree small linear openings (Fig. 7I)	***Nemorilla maculosa***

**Figure 12.**
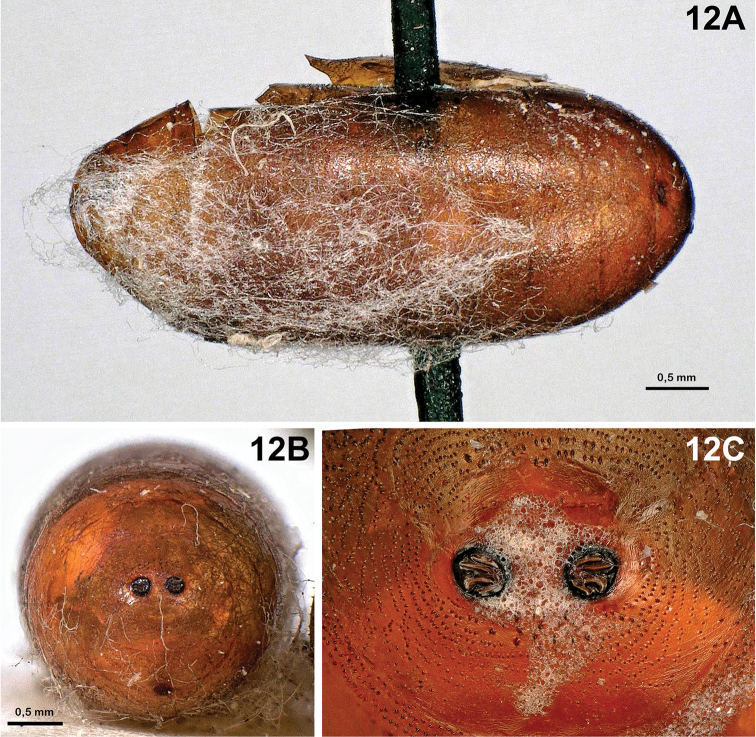
*Eurysthaea scutellaris* (Robineau-Desvoidy, 1848). **A** Puparium, lateral view **B** puparium, posterior end, posterior view **C** puparium, spiracular plates showing openings.

## Discussion

Both Lepidoptera and Tachinidae play a crucial role in agriculture and forestry, the first as pests and the second as potential BCAs. Therefore, information about parasitoid-host relationships may help in better understanding population dynamics of potential pests in different environments.

In this framework, we provided here eleven parasitoid-host records for tachinids in Italy and Spain. Some are new regional records for Italy, i.e., *Compsilura concinnata* on *Euproctis chrysorrhoea*, *Carcelia falenaria* on *Amata* sp., and Phryxe cf. nemea on *Tortrix viridana*, all collected on their hosts in Piedmont for the first time. *Pseudoperichaeta nigrolineata*, *Bessa parallela*, and *Nemorilla maculosa* are recorded for the first time in Italy on their renown hosts, i.e., *Cacoecimorpha pronubana*, *T. viridana*, and *Lobesia botrana* respectively. *Clausicella suturata* and *Neoplectops pomonellae* are reported for the first time on Ephestia unicolorella subsp. woodiella and *L. botrana*, respectively. The record of *Clemelis massilia* on *L. botrana* represents the first host record for this species so far.

Three out of these eleven species, Phryxe cf. nemea, *Bessa parallela*, and *Actia pilipennis*, have been obtained from *T. viridana*, one of the major defoliator pests of oaks in Europe, North Africa and Near East (Boghenschütz 1991). Five of these eleven species emerged from *L. botrana*, i.e., *C. massilia*, *N. maculosa*, *Phytomyptera nigrina*, *N. pomonellae*, and *Actia pilipennis*. So far, six species of tachinids have been associated with EGVM (Martinez et al. 2006, Delbac et al. 2015, Tschorsnig 2017), two of which (i.e., *P. nigrina* and *A. pilipennis*) have already been recorded on this pest in Italy (Scaramozzino et al. 2017a). Considering the present records as well as the one from Carlos et al. (2019), which confirms the previous observations made by Forti (in Coscollá 1997) and by Hoffman and Michl (2003), the number of tachinids associated with *L. botrana* rises to nine (Tab. 1). *P. nigrina* and *A. pilipennis* have been reared from *L. botrana* both in vineyards and on *Daphne gnidium* (Scaramozzino et al. 2017a), while the other three species, *C. massilia*, *N. maculosa* and *N. pomonellae*, have been obtained only from *D. gnidium* so far. In Spain, *P. nigrina* has been obtained from *L. botrana* in the vineyards (Coscollá 1981) and its presence into tortricid nests on *D. gnidium* in the wild is recorded here for the first time in the country. Among these nine species associated with *L. botrana*, *P. nigrina* certainly appears the most common and is also the most cited in the literature (see Tschorsnig 2017). All the other species seem to be occasional parasitoids, which also live at the expenses of other lepidopterans sharing the same host plant (Tab. 2). During our 3-year survey in the Natural Reserve of San Rossore (Tuscany, Italy), the overall parasitisation rate on preimaginal stages of *L. botrana* ranged between 12% and 16%, with tachinids accounting for 2–6% of the parasitoid community (Scaramozzino et al., unpublished data). In this context, they play a role as occasional parasitoids of *L. botrana* and other moths.

**Table 1.** Species of Tachinidae reported on *Lobesia botrana* in Europe. An asterisk indicates species previously reported on EGVM in Italy.

	Tachinid species	Main citations
1	*Actia pilipennis* (Fallén, 1810)*	Delbac et al. 2015, Scaramozzino et al. 2017a
2	*Bessa parallela* (Meigen, 1824) [as *Bessa selecta* in Jordan 1915 and Thomson 1946]	Tschorsnig 2017
3	*Clemelis massilia* Herting, 1977	Present paper
4	*Elodia morio* (Fallén, 1820)	Martinez et al. 2006, Tschorsnig 2017
5	*Eurysthaea scutellaris* (Robineau-Desvoidy, 1848)	Forti (as *Dischocaeta hyponomeutae*) in Coscollá 1997, Hoffman and Michl 2003, Carlos et al. 2019
6	*Nemorilla maculosa* (Meigen, 1824) (= *Nemorilla floralis* Fallén, 1810, misid.)	Martinez et al. 2006, Tschorsnig 2017
7	*Neoplectops pomonellae* (Schnabl & Mokrzecki, 1903)	Present paper
8	*Phytomyptera nigrina* (Meigen, 1824)*	Martinez et al. 2006, Tschorsnig and Cerretti 2010, Scaramozzino et al. 2017a, Tschorsnig 2017
9	*Pseudoperichaeta nigrolineata* (Walker, 1853)	Martinez et al. 2006, Tschorsnig 2017

**Table 2.** List of tachinid parasitoids and their related host species. Numbers indicate the total records reported in literature for each species (data from Tschorsnig 2017 and present paper).

Tachinid species	*Argyrotaenia ljungiana* (Thunberg, 1797)	*Cacoecimorpha pronubana* (Hübner, 1799)	*Cryptoblabes gnidiella* (Millière, 1867)	*Eupoecilia ambiguella* (Hübner, 1796)	*Lobesia botrana* (Denis & Schiffermüller, 1775)	*Sparganothis pilleriana* (Denis & Schiffermüller, 1775)
*Actia crassicornis* (Meigen, 1824)						1
*Actia pilipennis* (Fallén, 1810)		7			2	2
*Bessa parallela* (Meigen, 1824)	1	1		1	1	3
*Clemelis massilia* Herting, 1977					1	
*Elodia morio* (Fallén, 1820)					1	1
*Erynnia ocypterata* (Fallén, 1810)						6
*Eumea linearicornis* (Zetterstedt, 1844)						1
*Eumea mitis* (Meigen, 1824)						1
*Eurysthaea scutellaris* (Robineau-Desvoidy, 1848)				1	1	2
*Nemorilla floralis* (Fallén, 1810)	2	3				3
*Nemorilla maculosa* (Meigen, 1824)	1	1	1	1	4	6
*Neoplectops pomonellae* (Schnabl & Mokrzecki, 1903)					1	
*Pales pavida* (Meigen, 1824)		1				1
*Phytomyptera nigrina* (Meigen, 1824)				2	19	
*Pseudoperichaeta nigrolineata* (Walker, 1853)	1	1			1	7
*Pseudoperichaeta palesioidea* (Robineau-Desvoidy, 1830)		1				
*Thelyconychia solivaga* (Rondani, 1861)		1				
*Zenillia libatrix* (Panzer, 1798)	1					

## Supplementary Material

XML Treatment for Compsilura concinnata

XML Treatment for Carcelia falenaria

XML Treatment for Phryxe cf. nemea

XML Treatment for Pseudoperichaeta nigrolineata

XML Treatment for Bessa parallela

XML Treatment for Clemelis massilia

XML Treatment for Nemorilla maculosa

XML Treatment for Phytomyptera nigrina

XML Treatment for Clausicella suturata

XML Treatment for Neoplectops pomonellae

XML Treatment for Actia pilipennis
